# ^213^Bi-PSMA-617 targeted alpha-radionuclide therapy in metastatic castration-resistant prostate cancer

**DOI:** 10.1007/s00259-017-3657-9

**Published:** 2017-03-02

**Authors:** Mike Sathekge, Otto Knoesen, Marian Meckel, Moshe Modiselle, Mariza Vorster, Sebastian Marx

**Affiliations:** 1 Department of Nuclear Medicine, Steve Biko Academic Hospital, University of Pretoria, Pretoria, South Africa; 2 NTP Radioisotopes, The South African Nuclear Energy Corporation, Pelindaba, Pretoria, South Africa; 3 ITG Isotope Technologies Garching GmbH, Garching bei München, Germany


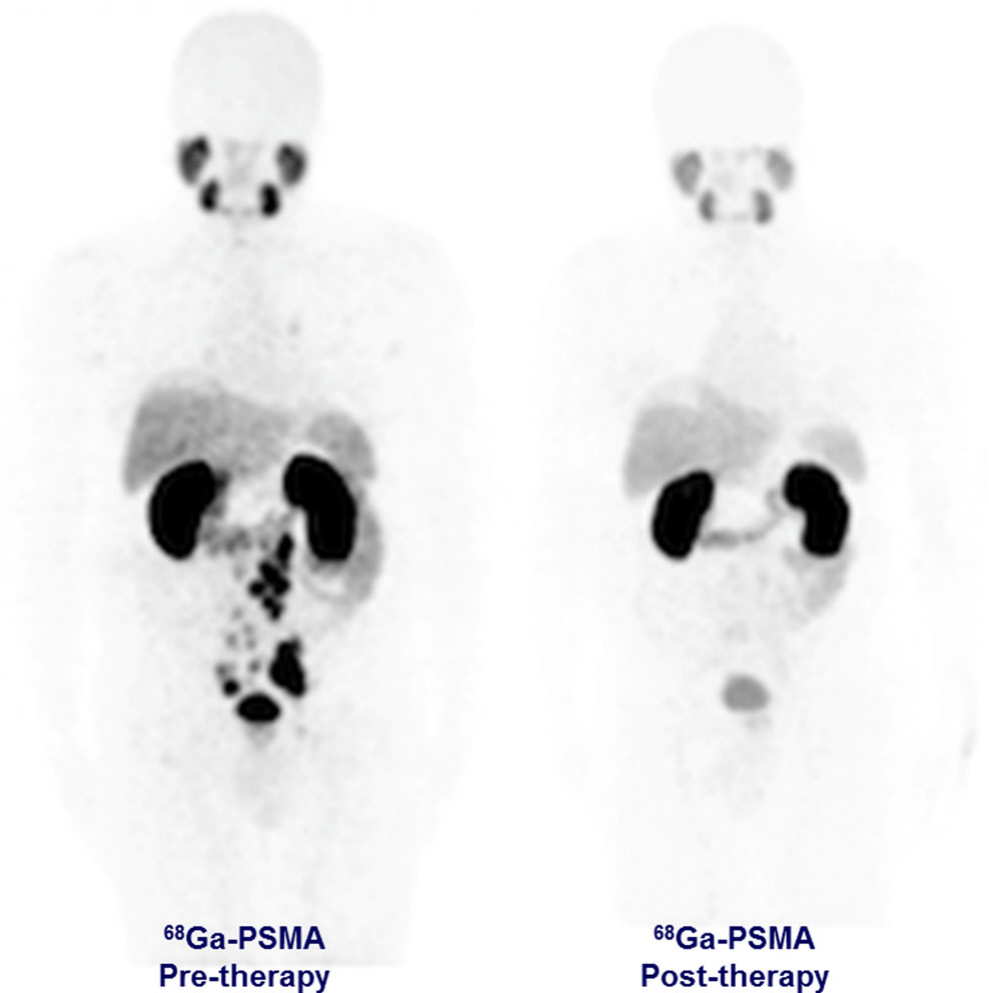


Prostate-specific membrane antigen radioligand therapy (PSMA-RLT) with ^177^Lu-PSMA holds great promise as a safe treatment option in patients with metastasized castration-resistant prostate cancer (mCRPC) with appropriate selection [1]. This approach, together with ^68^Ga-PSMA PET/CT, is an excellent example of theranostic nuclear medicine [2]. However, more structured data have recently shown that despite a marked response to PSMA-RLT, some patients are refractory to ^177^Lu-radioligand therapy [2, 3]. Fortunately recent studies have demonstrated that targeted α-radiation therapy with ^225^Ac-PSMA can significantly benefit mCRPC patients [4]. Similarly, ^213^Bi-DOTATOC may be able to break the radioresistance to β-emitters while simultaneously reducing haematological toxicity in patients with diffuse red marrow infiltration by neuroendocrine tumour [5].

We present the first-in-human treatment concept with ^213^Bi-PSMA-617 in a patient with mCRPC that was progressive under conventional therapy. The patient was treated with two cycles of ^213^Bi-PSMA-617 with a cumulative activity of 592 MBq. Restaging with ^68^Ga-PSMA PET/CT after 11 months showed a remarkable molecular imaging response. This patient also demonstrated a biochemical response (decrease in PSA level from 237 μg/L to 43 μg/L).

This case supports the need further exploration on the use and supply of targeted α-radiation therapy.
